# Nutritional Psychiatry: Knowledge, Attitudes and Clinical Practice of Mental Health Professionals

**DOI:** 10.1192/j.eurpsy.2022.1756

**Published:** 2022-09-01

**Authors:** P. Michielsen, D. De Smet

**Affiliations:** Mental Health Western Brabant, Outpatient Clinic, Halsteren, Netherlands

**Keywords:** Nutritional, prevention, treatment adherence, residency training

## Abstract

**Introduction:**

In 2016 the United Nations launched their “Decade of action on nutrition” promoting a healthy and sustainable food pattern. The International Society for Nutritional Psychiatry Research held its first International Conference in 2017. Current evidence in this area consists mainly of association studies, while interventional studies with food supplements or altered diet patterns are starting to emerge.

**Objectives:**

To our knowledge practice based research on promoting healthy food and investigating the role of medical professionals is scarse in general and especially so in psychiatry. Hence, our research questions were: 1. What is the attitude of mental health professionals with regard to promoting healthy food in their patients. 2. What is the subjective knowledge and attention in training schemes on this topic.

**Methods:**

We conducted a self-made online questionnaire using a 5 point Likert Scale. Surveys were sent out to 50 mental health professionals of our institution, including psychiatrists, psychiatric residents, General Practitioners and Mental Health Nurse Practitioners. Results were analysed with descriptive statistics.

**Results:**

40 (80%) of the respondents returned the questionnaire. 65% of respondents considered promoting healthy food as a key task for themselves in their daily practice. 45% of respondents believed their patients would be reluctant to follow advice on healthy diet. 62% had sufficient knowledge on the subject to give professional advice, while 65% answered this topic received insufficient attention during their training.

**Conclusions:**

In this survey we found the role of promoting healthy diet deserves more attention in mental health practice and training. Smartphone applications may ameliorate treatment adherence.

**Disclosure:**

No significant relationships.

